# Roles of Electrostatics and Conformation in Protein-Crystal Interactions

**DOI:** 10.1371/journal.pone.0009330

**Published:** 2010-02-19

**Authors:** Paul V. Azzopardi, Jason O'Young, Gilles Lajoie, Mikko Karttunen, Harvey A. Goldberg, Graeme K. Hunter

**Affiliations:** 1 School of Dentistry and Department of Biochemistry, University of Western Ontario, London, Ontario, Canada; 2 School of Dentistry, University of Western Ontario, London, Ontario, Canada; 3 Department of Biochemistry, University of Western Ontario, London, Ontario, Canada; 4 Department of Applied Mathematics, University of Western Ontario, London, Ontario, Canada; Massachusetts Institute of Technology, United States of America

## Abstract

*In vitro* studies have shown that the phosphoprotein osteopontin (OPN) inhibits the nucleation and growth of hydroxyapatite (HA) and other biominerals. *In vivo*, OPN is believed to prevent the calcification of soft tissues. However, the nature of the interaction between OPN and HA is not understood. In the computational part of the present study, we used molecular dynamics simulations to predict the adsorption of 19 peptides, each 16 amino acids long and collectively covering the entire sequence of OPN, to the {100} face of HA. This analysis showed that there is an inverse relationship between predicted strength of adsorption and peptide isoelectric point (P<0.0001). Analysis of the OPN sequence by PONDR (Predictor of Naturally Disordered Regions) indicated that OPN sequences predicted to adsorb well to HA are highly disordered. In the experimental part of the study, we synthesized phosphorylated and non-phosphorylated peptides corresponding to OPN sequences 65–80 (pSHDHMDDDDDDDDDGD) and 220–235 (pSHEpSTEQSDAIDpSAEK). In agreement with the PONDR analysis, these were shown by circular dichroism spectroscopy to be largely disordered. A constant-composition/seeded growth assay was used to assess the HA-inhibiting potencies of the synthetic peptides. The phosphorylated versions of OPN65-80 (IC_50_ = 1.93 µg/ml) and OPN220-235 (IC_50_ = 1.48 µg/ml) are potent inhibitors of HA growth, as is the nonphosphorylated version of OPN65-80 (IC_50_ = 2.97 µg/ml); the nonphosphorylated version of OPN220-235 has no measurable inhibitory activity. These findings suggest that the adsorption of acidic proteins to Ca^2+^-rich crystal faces of biominerals is governed by electrostatics and is facilitated by conformational flexibility of the polypeptide chain.

## Introduction

Biomineralization is the controlled deposition of crystals in tissues such as bones, shells and teeth. The hallmarks of biomineralization are precise control over crystal type, shape and orientation, as well as distinct spatial relationships between mineral and organic matrix [1]. In mammals, the mineral phase is almost invariably hydroxyapatite (HA; Ca_10_[PO_4_]_6_[OH]_2_). Ectopic calcification, the formation of crystals in soft tissues such as cartilage, kidney and blood vessels, is much less organized, often featuring variable crystal size, random orientation and no apparent matrix-mineral relationship. Hydroxyapatite (HA) occurs in calcified blood vessels (atherosclerosis), but many other mineral phases, including calcium oxalates (kidney stones) and uric acid (gout) are also found in calcified soft tissues.

Interactions between proteins and crystals are believed to play important roles in biomineralization [2]. Anionic proteins isolated from mineralized tissues have been shown to nucleate biomineral crystals [3], [4], [5], promote the formation of a particular polymorph [6], [7] or alter crystal growth habit [8], [9]. Protein-crystal interactions are also thought to prevent ectopic calcification [10]. Several proteins found in soft tissues or tissue fluids inhibit crystal nucleation and/or growth *in vitro* [11], [12]. Deletion of the genes encoding such proteins has been shown to result in organ-specific or systemic calcification [13], [14], [15], [16].

Like many crystal-inhibiting proteins, osteopontin (OPN) is found both in mineralized and nonmineralized tissues. *In vitro*, it has been shown to inhibit the formation of calcium phosphate, calcium oxalate and calcium carbonate crystals [17], [18], [19]. OPN is a phosphoglycoprotein of approximately 300 amino acids, many of which are aspartic or glutamic acid [20]. The extent of post-translational modification of the protein depends both on species and tissue of origin: cow milk OPN has 28 sites of phosphorylation [21], with an average phosphate content per molecule of 25 [22]; while rat bone OPN has 29 sites of phosphorylation, with an average phosphate content of 10 [23].

Phosphate groups present in OPN make a large contribution to the crystal-inhibiting activity of the protein. Thus, nonphosphorylated forms of OPN or OPN peptides are far less inhibitory than the corresponding phosphorylated protein/peptide [9], [18], [24], [25], [26]. However, it is not clear whether or not highly phosphorylated forms of OPN like that from breast milk are significantly better inhibitors than less-phosphorylated forms like that from bone [22], [27]. Also, it appears that some OPN phosphopeptides are stronger inhibitors than others of similar phosphate content [28], [29].

The means by which OPN inhibits the formation of biominerals is also unclear. Studies of calcium oxalate monohydrate (COM) growth using atomic force microscopy have shown that growth-hillock structure is disrupted and the rate of step growth decreased in the presence of OPN [30]. This has been interpreted in terms of a step-pinning mechanism in which the adsorption of a sufficient number of OPN molecules to growth steps prevents the steps from advancing over the crystal face. These studies have also shown that OPN has a preference for certain types of steps on the COM crystal [30]. Specificity of interaction is also suggested by our previous demonstration that a synthetic phosphopeptide corresponding to amino acids 220-235 of rat bone OPN adsorbs selectively to {100} faces and preferentially inhibits growth in <100> directions (perpendicular to {100} faces) [9]. The selectivity of OPN220-235 for the {100} face appears to be due to the electropositivity of this face compared to the other faces developed ({010} and {121}) [31].

Far less is known about the interaction between OPN and HA. Largely this is because most synthetic and biological HA crystals have dimensions in the order of tens of nanometres – almost three orders of magnitude smaller than COM crystals.

The lack of experimental approaches to study HA crystal growth can be to some extent remedied by the use of molecular-dynamics simulations [32]. In previous studies, we have used molecular dynamics to study the interaction between the OPN220-235 peptide and the {100} face of COM. This showed that the amino acids interacting most closely with the face are aspartic and glutamic acids, not phosphoserines, and also provided information about the positions of carboxylate and phosphate oxygen atoms relative to the Ca^2+^ ions of the {100} face [9], [33].

For the purposes of the present study, we have now created a molecular-dynamics simulation of the {100} face of HA. This is the principal crystal face developed in bioapatites and has been implicated in the adsorption of other acidic proteins [34], [35], [36], [37]. Using this {100}-face simulation, we have studied the interactions of a series of virtual peptides covering the entire sequence of rat bone OPN. To validate the results of the simulations, we synthesized a peptide predicted to interact strongly with the HA {100} face and showed, using a constant-composition assay, that this peptide is a potent inhibitor of the growth of HA crystals. The results obtained from this study allow us to describe the roles of charge and conformation in the interaction between OPN and HA.

## Materials and Methods

### Molecular-Dynamics Simulations

Atomic-scale molecular-dynamics simulations were performed using the GROMACS suite [38]. For force field, we used GROMOS96 version 45A3, which has proven to be a reliable description for lipids, peptides and other biomolecules [39]. Similar methods and software were used in a previous study of HA-water interactions [40]. Other studies on HA and related crystals have used the CHARMM [41], [42] or COMPASS [43] force-fields, as well as a number of individual parameterizations.

The coordinates for the HA {100} face were taken from previously obtained experimental results [44]. The topologies for the phosphate and hydroxyl ions was generated using previously solved atomic charges [45], [46] and parameters from the force field for constraints. Note that our HA simulation does not include the kinds of imperfections (disclocations, vacancies, step edges, etc.) that occur in “real” crystals, as these would greatly complicate the analysis. Simulations by other workers also involve perfect crystal lattices (for review, see [32], [47]).

Extended conformations were used as the initial peptide structure. For each simulation, peptides were oriented parallel to the crystal surface where the center-of-mass difference between the crystal slab and the peptide was approximately 4 nm in the direction perpendicular to the surface. The crystal slab was placed at the center of the periodic cell and constructed to be approximately 1.0 nm thick with the Ca^2+^-dense layers of the {100} face exposed on each side. The simulations were performed in the NVT ensemble at 300 K and periodic boundary conditions were applied with the size of the simulation cell being 8.4 nm×6.2 nm in the plane of the surface and 10 nm perpendicular to the surface. The system was solvated with simple point charge (SPC) water [48] model which is consistent and proven to work well with the GROMOS96 force field [49]. Cl^-^ counter-ions were added to maintain the system charge-neutral. Prior to the actual simulation runs, energy minimization was performed without constraints using the steepest descent method.

The bond lengths were constrained using the SHAKE algorithm [50]. Crystal atoms were constrained to their equilibrium positions. 1.0 nm cutoff was used for the Lennard-Jones interactions as required by the chosen force-field. The weak-coupling thermostat with a coupling time constant of 0.1 ps was employed and the particle mesh Ewald method [51], [52], [53] with real space cutoff of 1.0 nm, beta-spline interpolation of order 6 and direct sum tolerance of 10^−6^ was used for electrostatics. Since the system contains strong charges, it is important to employ proper treatment of electrostatics (for a comprehensive discussion see [54]) as cutoffs have been shown to lead to significant artifacts in biomolecular simulations [52]. The time step was set to 2 fs, which is the standard when no driving forces, such as shear, are present. Systems were simulated for 5 ns each. The systems consisted of total of 49,438–49,485 atoms. The number of water molecules was about 14,250, varying slightly depending on the system. All simulations were run in parallel over eight processors on the SHARCNET grid computing facility (www.sharcnet.ca). To reduce potential bias due to initial conditions, 6 different initial conditions were used in all of the cases. In total, 74 simulation runs were performed.

Distance from the crystal surface for each peptide was calculated by averaging the center-of-mass position in the vertical axis of the simulation box over 3 to 5 ns sampled at 20 ps intervals. The vertical position of the crystal surface atoms was subtracted from this value to arrive at the final result.

### Calculation of Peptide Isoelectric Points

Isoelectric points of OPN virtual peptides were determined using the calculator developed by Gauci and coworkers. This instrument calculates the pI of a peptide at a particular pH using user-specified p*K* values. The calculation is repeated until the pH corresponding to a net charge of zero is found [55]. pI values quoted were calculated using the Scansite and Expasy options.

### Synthesis and Characterization of Peptides

OPAR (osteopontin poly-aspartate region: SHDHMDDDDDDDDDGD) and pOPAR (pSHDHMDDDDDDDDDGD) peptides were synthesized by a batch method with free amino and carboxyl termini using Fmoc chemistry and purified by high-performance liquid chromatography on a C18 column, as previously described [9], [29]. Peptide purity was determined by electrospray ionization mass spectrometry (OPAR, 1,833.29 Da; pOPAR, 1913.13 Da) and amino acid analysis (Institute for Biomolecular Design, University of Alberta, or Advanced Protein Technology Centre, Hospital for Sick Children, Toronto). The P0 (SHESTEQSDAIDSAEK) and P3 (pSHEpSTEQSDAIDpSAEK) peptides were those previously described [9].

Circular dichroism studies were performed using a Jasco J-810 spectropolarimeter equipped with a Peltier temperature-control system. Each peptide was resuspended at a concentration of 0.4 mM in either Ca/PO_4_ [500 µM Ca(NO_3_)_2_, 300 µM Na_2_HPO_4_, 150 mM NaCl, pH 7.4] or HEPES (10 mM HEPES, 100 mM NaCl, 10 mM KCl, pH 7.4) buffer. Scans were recorded at 37°C from 250 to 190 nm, with a step size of 0.5 nm and a scan speed of 100 nm/min. A cell with a path length of 0.1 mm was used. Each peptide solution was scanned 30 times and the resulting spectra averaged. Blank buffer scans were subtracted from the raw data, which were then converted to mean residue ellipticity (θ) in units of degree cm^2^ dmol^−1^ by standard procedures. CDSSTR and CONTINLL algorithms for the estimation of protein secondary structure from UV CD spectra were used to analyze the circular-dichroism spectra generated [56].

### Constant-Composition/Seeded-Growth Analyses

HA seed crystals were prepared essentially by the method of Nancollas and Mohan [57] and characterized by X-ray diffractometry. Using the Brunauer-Emmett-Teller method, the surface area of the crystals was shown to be 84.1±0.094 m^2^/g.

A modification of the constant-composition seeded-growth assay originally developed by Tomson and Nancollas was used [58]. Reaction solutions were prepared by combining 1.25 ml of dH_2_O (or protein/peptide dissolved in dH_2_O), 2 ml of 1.2 mM Na_2_HPO_4_ and 4 ml of 1 mM Ca(NO_3_)_2_/300 mM NaCl in a custom-made double-walled Pyrex vessel with stirring. The solution was maintained at 37±0.1°C using a circulating water bath connected to the Pyrex vessel. All stock solutions were previously vacuum-filtered through 0.2 µm-pore-size polyethersulfone membranes. A calomel pHC4006 electrode connected to a TIM900 titration manager (Radiometer) was immersed into the reaction solution. To exclude atmospheric carbon dioxide, a single flow tube rotameter was used to bubble 18.3 ml/min of water-saturated nitrogen through the solution. To provide adequate time to reach equilibrium, nitrogen flow began one hour prior to the addition of seed crystals. Prior to the addition of HA seed crystals, the pH of the metastable solution was adjusted to between 7.40 and 7.41 by the addition of small aliquots of 25 mM NaOH.

Also immersed in the reaction solution were two Teflon titration probes attached to a Radiometer ABU93 triburet. The triburet was customized so that two of its 5-ml burets operated in the “master-slave” mode. The “master” buret contained 3.5 mM Ca(NO_3_)_2_/300 mM NaCl and the “slave” buret contained 2.1 mM Na_2_HPO_4_/1.6 mM NaOH. Titrant addition was controlled using TimTalk 9 in pH-stat mode with an endpoint pH of 7.40 and proportional band pH of 0.100. The burets were limited to a minimum speed of 1.0% volume/min and a maximum speed of 3.0% volume/min.

The reaction was initiated by the addition of 750 µl of a freshly made HA slurry in dH_2_O (2.67 mg/ml, unless otherwise stated). The final reaction solution composition was therefore 0.5 mM Ca(NO_3_)_2_, 0.3 mM Na_2_HPO_4_, 150 mM NaCl and 0.25 mg/ml HA.

Immediately after the addition of the hydroxyapatite slurry (time zero) and just prior to the termination of the titration (240 min), 0.4-ml aliquots were removed from the reaction solution and filtered through 0.2-µm polyethersulfone membrane syringe filters. The calcium and phosphate concentrations were determined spectrophotometrically using the QuantiChrom™ Calcium Assay Kit and the Innova Biosciences PiColorLock™ Phosphate Assay Kit according to the manufacturers' instructions.

## Results

### Molecular-Dynamics Analysis of Peptide-Hydroxyapatite Interaction

**Figure 1:**
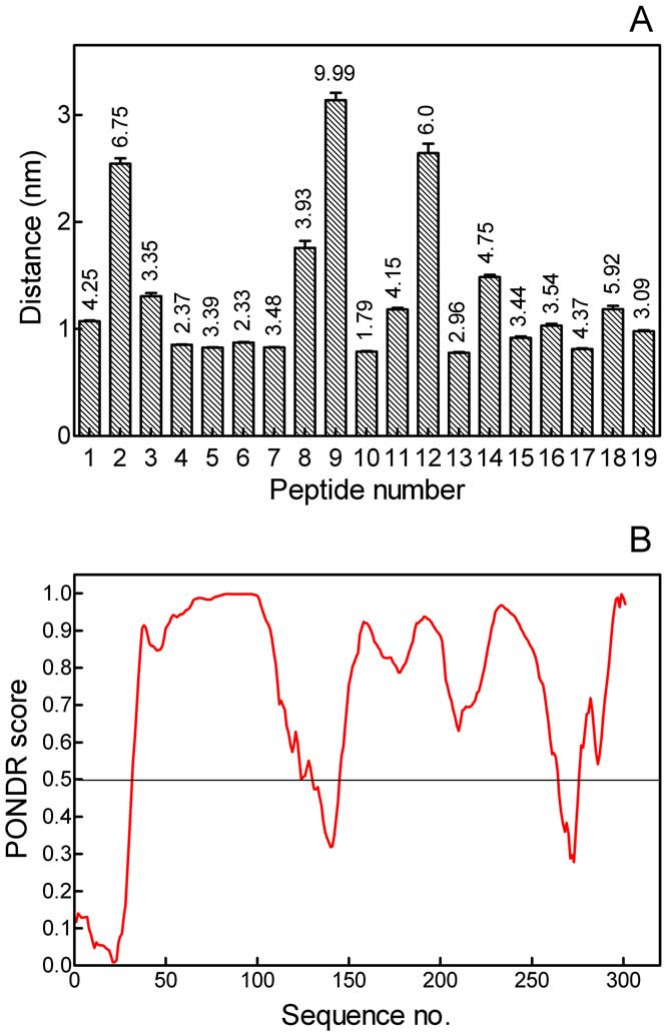
Molecular-dynamics analysis of OPN adsorption to HA and PONDR analysis of OPN structure. A. Distances between centers of mass of OPN virtual peptides (see Table 1) and outermost atoms of the {100} face of HA. Error bars represent root mean square deviations of peptide-crystal distance over 3–5 nsec of simulation. Numbers above bars are the isoelectric points of the peptides. B. PONDR analysis of the primary sequence of rat OPN. A score of >0.5 is indicative of a disordered sequence.

The rat bone OPN sequence was divided into 19 virtual peptides, each 16 amino acids long. The sequences of these peptides are shown in Table 1. Note that, because the number of amino acids in rat OPN (301) is not an exact multiple of 16, peptides 18 and 19 overlap by three amino acids. Each peptide was placed in a simulation box containing a section of the {100} face of HA, Cl^−^ counterions and water and subjected to a molecular-dynamics force-field for 5 ns of simulation time. At the end of the simulations, the final distance between the peptide center of mass and the outermost layer of crystal atoms was calculated (Figure 1A). It can readily be seen that the peptides forming close contact with the {100} face are those with low isoelectric points, while the three peptides with near-neutral or basic isoelectric points are by far the most distant from the face. No peptide has a center-of-mass distance less than approximately 0.8 nm, which probably represents the closest contact between peptide and crystal that does not infringe upon the van der Waals' radii of any atom.

**Table 1:** Amino acid sequence and pI of virtual OPN peptides used for MD analysis.

Peptide no.	Amino acid nos.	Sequence	Isoelectric point
1	1–16	LPVKVAEFGpSpSEEKAH	4.25
2	17–32	YSKHSDAVATWLKPDP	6.75
3	33–48	SQKQNLLAPQNSVpSpSE	3.35
4	49–64	EpTDDFKQETLPpSNpSNE	2.37
5	65–80	pSHDHMDDDDDDDDDGD	3.39
6	81–96	HAEpSEDSVNpSDEpSDES	2.33
7	97–112	HHpSDEpSDESFTASTQA	3.48
8	113–128	DVLTPIAPTVDVPDGR	3.93
9	129–144	GDSLAYGLRSKSRSFP	9.99
10	145–160	VpSDEQYPDApTDEDLTpS	1.79
11	161–176	RMKpSQEpSDEALKVIPV	4.15
12	177–192	AQRLSVPSDQDSNGKT	6
13	193–208	pSHEpSSQLDEPpSVETHS	2.96
14	209–224	LEQSKEYKQRApSHEpST	4.75
15	225–240	EQSDAIDpSAEKPDAID	3.44
16	241-256	pSAERpSDAIDSQASSKA	3.54
17	257–272	pSLEHQpSHEFHpSHEDKL	4.37
18	273–288	VLDPKpSKEDDRYLKFR	5.92
19	286–301	KFRIpSHELEpSpSpSSEVN	3.09

Underlined amino acids in peptides 14 and 15 correspond to the sequence of P3.

**Figure 2:**
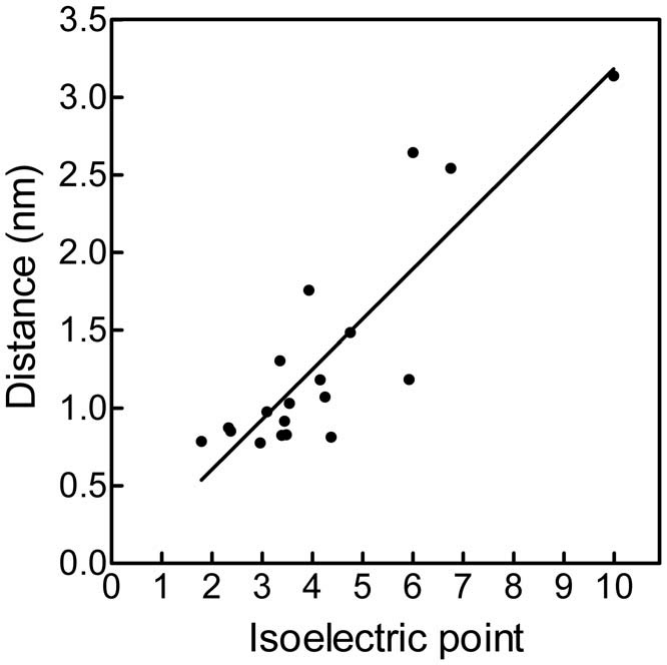
Relationships between peptide isoelectric point and predicted adsorption to HA. Peptide-crystal distances and isoelectric points are from Figure 1. Equation of regression line is *y* = 0.323 *x*– 0.040 (r^2^ = 0.754, P < 0.0001).

In Figure 2, distance between the peptide center of mass and the outermost layer of crystal atoms is plotted against isoelectric point. There is a statistically significant correlation between distance and pI, such that peptides with lowest pIs approach closest to the {100} face. This correlation implies that the nature of the amino acids contributing to the negative charge (aspartic acid, glutamic acid, phosphoserine, phosphothreonine) of the peptide is relatively unimportant.

The degree of molecular order of the rat OPN sequence was analyzed using PONDR (Predictor Of Naturally Disordered Proteins, www.pondr.com) [59]. As this neural network cannot account for post-translational modifications, the sequence analyzed was the primary structure of the protein. A PONDR score of greater than 0.5 is considered to indicate disorder. The PONDR analysis of OPN is shown in Figure 1B. Two features are obvious. First, rat OPN is highly disordered, with the great majority of the sequence having PONDR scores much greater than 0.5. The only ordered regions are amino acids 1–31, 131–144 and 265–275. Second, there is generally an inverse relationship between PONDR score and peptide distance from the {100} face of HA. For example, peptides 3–7, which are predicted by molecular dynamics to interact strongly with the crystal face, are predicted by PONDR to be highly disordered, while peptide 9 is predicted to interact poorly and be relatively ordered.

**Figure 3:**
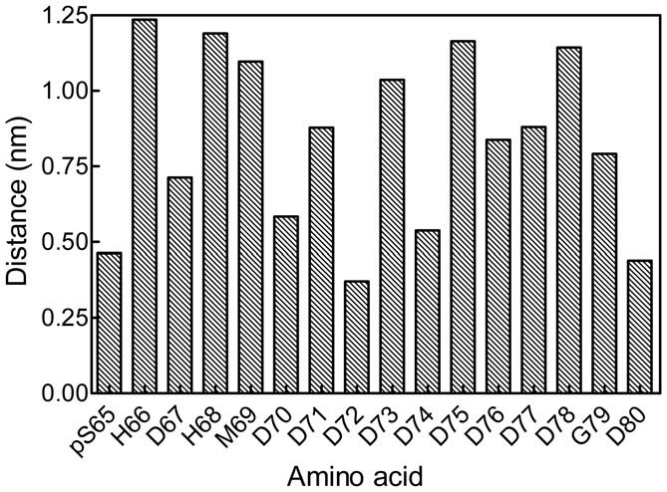
Molecular-dynamics analysis of pOPAR adsorption to HA. Distances between pOPAR side-chain centres of mass and outermost atoms of the {100} face of HA. Distances were averaged over 3–5 nsec of simulation time.

**Figure 4:**
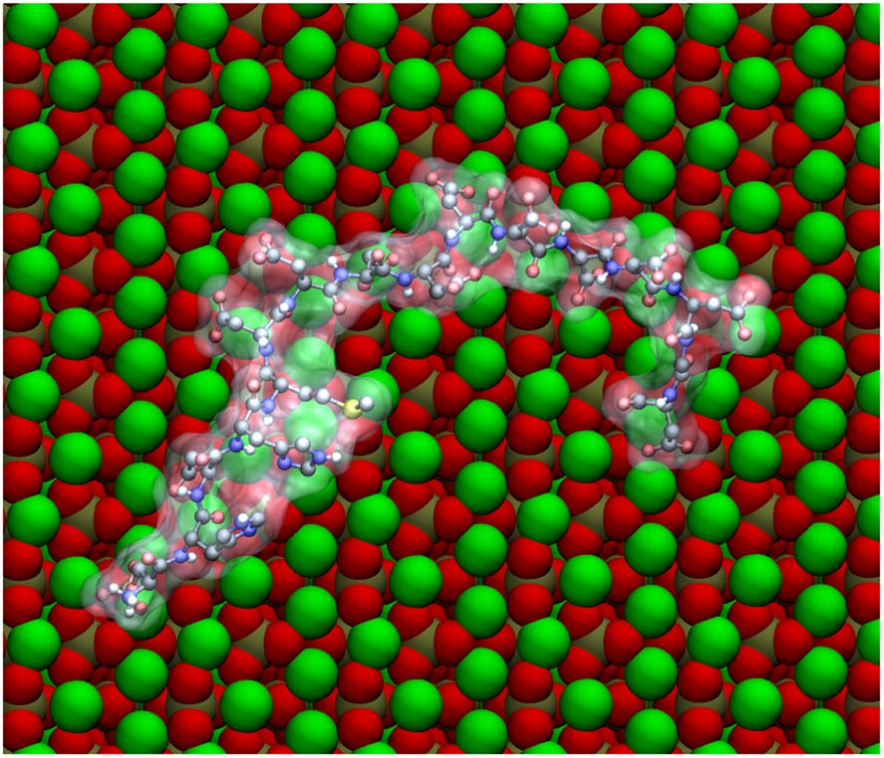
Orientation of pOPAR on the {100} face of HA. Peptide is viewed at the end of the 5-ns simulation. Crystal: Ca – green, O – red, P – orange. Peptide: C – grey, H – white, O – pink, N – purple, P – orange, S – yellow.

One of the virtual OPN peptides predicted by molecular dynamics to adsorb most closely with the {100} face of HA is 65–80, pSHDHMDDDDDDDDDGD, which contains the poly-aspartic sequence of the protein. A movie of the molecular-dynamics simulation of the interaction of this peptide with the {100} face is presented as Movie S1. Distances between the side-chain centers of mass of OPN65-80 and the outer layer of crystal atoms were averaged over the period 3–5 ns of simulation time (Figure 3). The amino acids furthest from the crystal face include the slightly cationic histidines and the bulky methionine. Those closest to the face are the single phosphoserine and two aspartic acids. Generally speaking there is an alternation of closer and more-distant amino acids along the sequence of the peptide. The final (5-nsec) conformation of OPN65-80 is viewed perpendicular to the {100} face in Figure 4. The peptide backbone is not straight, and therefore is not aligned with any row of Ca^2+^ ions in the {100} plane.

**Figure 5:**
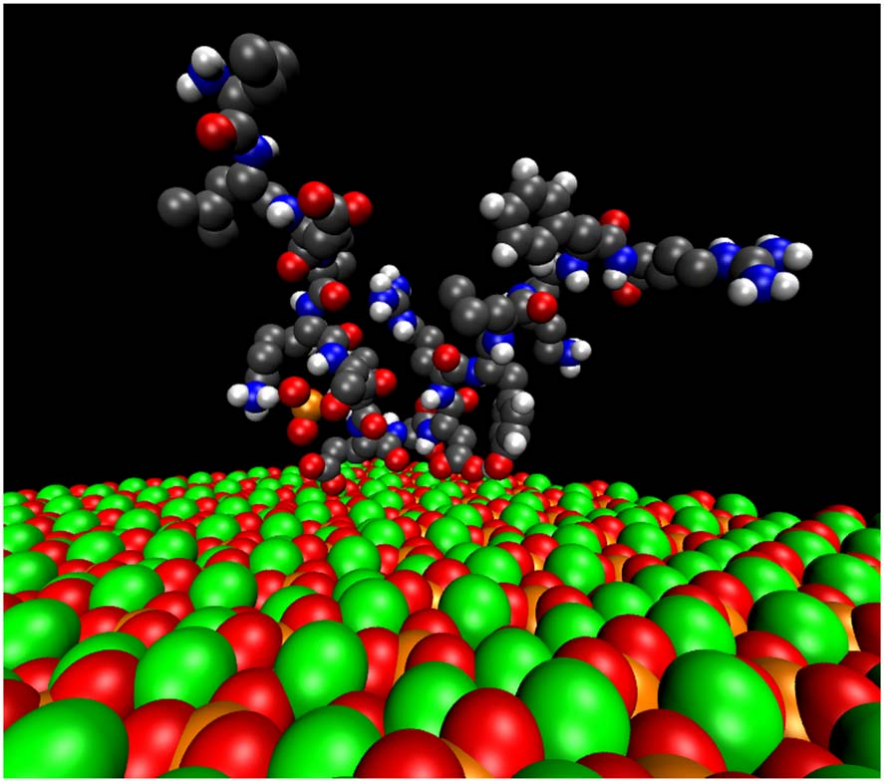
Molecular-dynamics analysis of peptide VLDPKpSKEDDRYLKFR adsorption to HA. Peptide is viewed at the end of the 5-ns simulation. Ca – green, O – red, P – orange, C – grey, H – white, N – blue.

Virtual peptide VLDPKpSKEDDRYLKFR (peptide 18 in Table 1) exhibits anomalous predicted adsorption behavior, as its center-of-mass distance from the {100} face is lower than its isoelectric point (5.92) would suggest. Interestingly, this peptide has the highest content of basic amino acids (five). The final (5-nsec) conformation of VLDPKpSKEDDRYLKFR is shown in Figure 5. Interaction of the peptide with the {100} face involves the central acid amino acids (EDD), while the more basic and hydrophobic N- and C-terminal ends do not form attachments with the crystal.

### Secondary Structures of Osteopontin Peptides

Synthetic peptides corresponding to amino acids 65–80 of rat bone OPN, with or without a phosphate group on the N-terminal serine, were generated. The non-phosphorylated version is referred to below as OPAR and the phosphorylated version as pOPAR. The secondary structures of these synthetic peptides were analyzed by circular dichroism spectrapolarimetry. Also studied were the P0 and P3 peptides, corresponding to amino acids 220–235 of rat bone OPN with or without the three phosphate groups present in this sequence. The P3 sequence does not correspond to any one of the virtual peptides analyzed by MD in this study; rather, it is divided between peptides 14 and 15 (Table 1). Circular dichroism spectra of these peptides, collected in either HEPES buffer or calcium phosphate solution, are shown as Figure S1. Deconvolution of the spectra with the CDSSTR and CONTINLL algorithms resulted in the secondary-structure contents shown in Table 2. For both peptides, there is very little difference between the solutions used. In general, the predicted α-helix content is very small, there is some β-turn and the highest percentage of ordered structure is β-strand. For OPAR and pOPAR, approximately 50% of the peptide is predicted to be unordered; for P0 and P3, approximately 70% is unordered.

**Table 2:** Secondary Structure Compositions of Synthetic OPN Peptides.

Peptide	Buffer	α-helix (%)	β-strand (%)	β-turn (%)	unordered (%)
OPAR	HEPES	3.5	23.1	13.1	59.7
	CaPO_4_	4.1	27.6	14.6	53.0
pOPAR	HEPES	2.4	30.4	17.1	49.2
	CaPO_4_	3.7	28.9	18.0	48.6
P0	HEPES	3.1	17.3	9.6	69.8
	CaPO_4_	3.6	12.2	7.4	76.1
P3	HEPES	2.3	15.9	9.2	72.1
	CaPO_4_	3.6	15.2	9.2	71.5

The percent compositions are derived from the circular dichroism spectra shown in Figure S1 using the CDSSTR and CONTINLL algorithms.

### Inhibition of Hydroxyapatite Growth by Osteopontin Protein and Peptides

**Figure 6:**
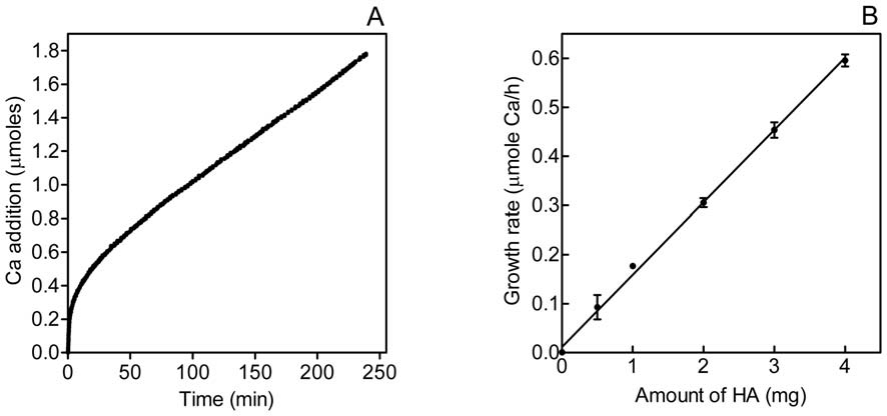
Constant-composition/seeded growth assay of HA formation. A. Typical titration curve obtained in the absence of effector. B. Relationship between rate of titrant addition and amount of seed crystal added. Equation of regression line is *y* = 0.147 *x* + 0.012 (r^2^ = 0.988, P < 0.001).

The effects of osteopontin peptides on HA formation were studied using a constant-composition/seeded-growth assay. In this assay, HA seed crystals are grown in a metastable calcium phosphate solution and a pH electrode is used to control the addition of titrant solutions containing the crystal lattice ions (Ca^2+^, PO_4_^3−^ and OH^−^). If the ratio of ions in the titrants corresponds to the ratio of ions incorporated into the crystal, the ionic composition of the solution will remain constant. To ensure that this was the case, Ca^2+^ and phosphate concentrations were measured at the beginning and end of the incubation. If the difference was greater than 5%, the experiment was discarded. Under the conditions used, the growth of the crystals is hyperbolic for approximately 60 min and linear thereafter (Figure 6A). The slope of the linear part of the growth curve is proportional to seed-crystal weight over the range 0.5–4 mg with a slope of unity; that is, doubling of the amount of crystal results in doubling the rate of titrant addition (Figure 6B).

**Figure 7:**
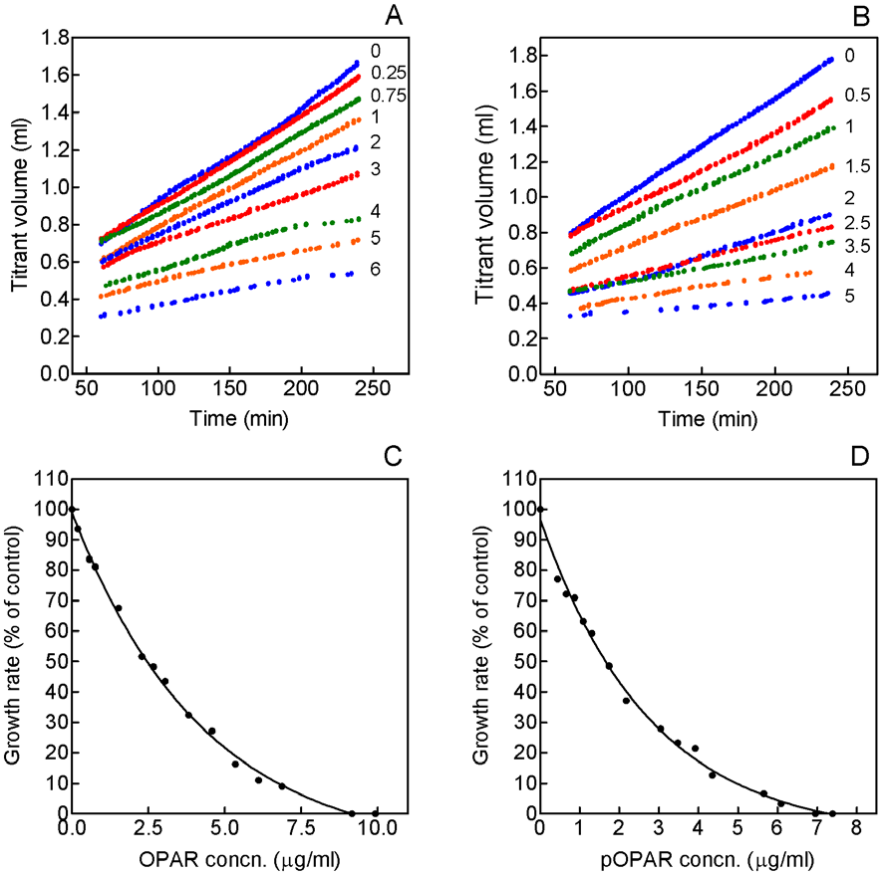
Effects of OPAR and pOPAR on seeded growth of HA. A. Titration curves obtained in the presence of OPAR. Nonlinear parts of the curves (0–60 min) have been omitted. Labels represent concentration in µg/ml. B. Plot of HA growth rate (see panel A) against OPAR concentration. The half-life of the one-phase exponential-decay curve (IC_50_) is 2.97. C. Titration curves obtained in the presence of pOPAR. Nonlinear parts of the curves (0–60 min) have been omitted. Labels represent concentration inµg/ml. B. Plot of HA growth rate (see panel C) against pOPAR concentration. The half-life of the one-phase exponential-decay curve (IC_50_) is 1.93.

**Figure 8:**
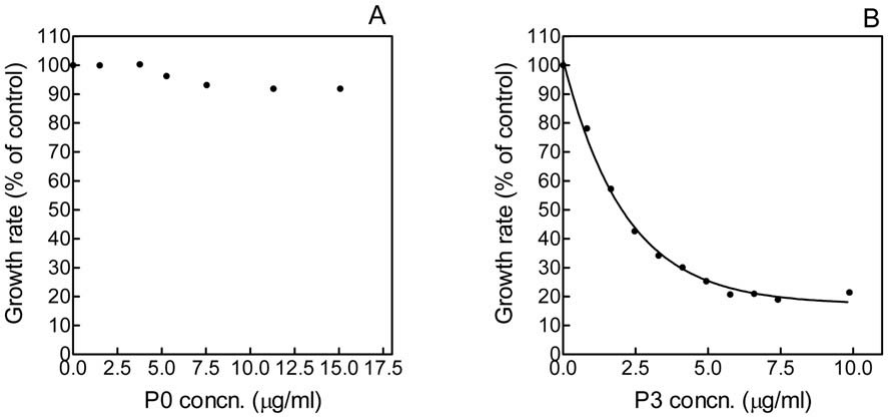
Effects of P0 and P3 on seeded growth of HA. A. Plot of HA growth rate against P0 concentration. B. Plot of HA growth rate against P3 concentration. The half-life of the one-phase exponential-decay curve (IC_50_) is 1.48.

Addition of OPAR or pOPAR causes a dose-dependent decrease in crystal growth rate (Figure 7A, B). Plots of growth rate against peptide concentration fit well to exponential-decay curves, with complete inhibition of growth occurring at the higher concentrations of peptide used (Figure 7C, D). From these curves, the following IC_50_ values were calculated: OPAR, 2.97 µg/ml and pOPAR, 1.93 µg/ml. Inhibition of HA growth by P0 and P3 was also studied (Figure 8). P0 was a very poor inhibitor, resulting in a less-than-10% decrease in titrant addition at a peptide concentration of 15 µg/ml (Figure 8A). Consequently, no IC_50_ value could be determined. P3 caused a dose-dependent decrease in crystal growth rate, corresponding to an IC_50_ of 1.48 µg/ml (Figure 8B). Unlike OPAR and pOPAR, however, the P3 inhibition curve did not decrease to zero, instead reaching a plateau value of approximately 20% of the control rate.

IC_50_ data are compared with pI values in Table 3.

**Table 3:** Inhibitory potencies of osteopontin peptides.

Peptide	pI	IC_50_ (µg/ml)	IC_50_ (µM)
OPAR	3.60	2.97	1.62
pOPAR	3.39	1.93	0.867
P0	4.17	>75	>42.6
P3	2.92	1.48	0.750

Isoelectric point of pOPAR is from Table 1. Isoelectric points of OPAR, P0 and P3 were derived as described in Experimental Procedures. IC_50_ values were derived from the data shown in Figures 7 and 8.

## Discussion

Because of the lack of physical methods capable of providing suitable resolution, simulation techniques are increasingly being used to study adsorption of biomolecules to crystals. In the case of HA, density-function methods have been used to study the adsorption of amino acids [60], energy minimization to study the adsorption of citrate [61] and molecular dynamics to study the ordering of water molecules [40]. A few studies have modelled the interactions of proteins or peptides with HA. Using energy minimization, it was shown the salivary protein statherin adsorbs equally well to {001}, {010} and {100} faces of HA. This interaction involved acidic and basic amino acids near the N-terminus of the protein [62]. Energy minimization has also been used to model the adsorption to HA of phosphopeptides typical of mineralized-tissue proteins. Oligomers containing phosphoserine-aspartic acid dipeptides were reported to interact favourably with {001} faces of HA [63]. However, a pentapeptide of phosphoserine and glutamic acid was found to adsorb to {010} and {100} faces in preference to {001} faces [36]. Pan and co-workers used a combination of molecular dynamics and steered molecular dynamics to study the adsorption of fibronectin and bone morphogenetic protein 2. In both cases, carboxylate, amino and hydroxyl groups were involved in the interaction of the protein with {001} faces of HA [64], [65].

As MD is computationally very intensive, analysis of interactions involving intact proteins at atomic scale is usually not practical. For a protein like OPN that has substantial sequence redundancy and lack of secondary/tertiary structure, one solution is to divide the protein into virtual peptides, many of which have similar motifs, and analyze the interaction with a crystal face of each peptide separately. This is the approach that we have developed to study the interaction between rat bone OPN and the {100} face of HA. To avoid bias, the peptides were created simply by dividing the 301-amino-acid sequence into 16-amino-acid segments starting at the N-terminus (except for a 3-amino-acid overlap between peptides 18 and 19), and therefore do not correspond to known degradation products of rat bone OPN or to predicted proteolytic cleavage sites.

Because of the large number of peptides to be analyzed, the simulations here are relatively short in duration (5 ns). This simulation time is, however, comparable to or longer than those used recently in similar studies that produced robust results and reliable comparisons with experimental findings. This is particularly the case for the quantities studied here, as formation of contacts between the protein and the surface occurs in short time-scales (<1 ns). In view of the rapid adsorption and multiple bonds formed, it is unlikely that desorption or conformational change will occur over time-scales achievable in simulation. The effects of initial peptide orientation on adsorbed conformation will be studied in detail in a separate publication. This will involve also an analysis of commensurability effects and free energy.

The 19 virtual peptides of OPN exhibit large differences in predicted HA-binding behavior, their center-of-mass distances from the {100} face at the end of the simulation ranging from about 0.8 nm to just over 4 nm. Those adsorbing most closely to the crystal have the lowest isoelectric points; those adsorbing least well have the highest isoelectric points. The correlation between OPN-peptide net charge and predicted strength of interaction with the crystal surface suggests that the adsorption of OPN peptides, almost all of which are acidic, to the basic {100} face of HA is governed by electrostatics. Based on their studies on the interaction between a phosphopeptide corresponding to amino acids 93–106 of human OPN and the {100} face of COM, a similar conclusion was reached by Wang et al [26]. Electrostatics has been reported to dominate in the adsorption to HA of peptides of β-casein [36], fibronectin [65] and statherin [66]. Our recent analysis of the effects of urinary proteins and model compounds on calcium oxalate crystal formation also shows that the most potent inhibitors have high negative charge density and high hydrophilicity [67]. These factors have also been implicated in the enhancement of calcite growth by acidic peptides [68].

The PONDR analysis reported here shows that OPN is unordered over more than 80% of its sequence. In fact this is probably an underestimate, as the analysis was performed on the primary sequence of rat OPN, and post-translational modification will likely decrease the order further. The high PONDR scores associated with most of the OPN sequence should come as no surprise, since it has all the hallmarks of an intrinsically unordered protein: a high content of charged amino acids, a low content of hydrophobic amino acids and a high degree of sequence redundancy [69]. In addition, OPN has been shown to lack folded structure by NMR spectroscopy and infrared spectroscopy [27], [70]. As shown here and previously by others [26], [28], synthetic peptides corresponding to sequences of OPN are largely unordered. These findings support suggestions by others that the flexible conformations of crystal-modulating phosphoproteins facilitate their interactions with biominerals [70], [71].

Comparison of the PONDR analysis of OPN and the molecular-dynamics analysis of adsorption to the {100} face of HA shows that strongly interacting regions of the protein tend to be highly disordered, whereas poorly interacting regions tend to be relatively ordered. This appears to imply a causal relationship between molecular disorder and ability to inhibit crystal growth. However, the inverse relationship between PONDR score and peptide-crystal distance may simply reflect the fact that high negative charge density contributes to molecular disorder by intramolecular electrostatic repulsion. Thus, it may be the charge density, not the consequent disorder, that determines adsorption strength and inhibition potency.

One of the virtual peptides predicted to adsorb best to the {100} face of HA is pSHDHMDDDDDDDDDGD (pOPAR), which is highly electronegative due to the fact that it contains the so-called “poly-aspartic acid” region of OPN. When the sequence of OPN was first determined, this region was immediately proposed to be the HA-binding site [20], although it was many years before any evidence in support of this proposal was obtained [72]. The predicted conformation of pOPAR at the end of the MD simulations described above shows that the peptide is not aligned with the principal rows of Ca^2+^ in the {100} plane, which run parallel to the crystallographic *c* axis. Indeed, the peptide backbone exhibits several bends, resulting in the N-terminal and C-terminal portions being approximately antiparallel. Non-linear conformations of crystal-bound peptides have also been predicted for a lithostatine undecapeptide adsorbed to calcite [73] and a dentin matrix protein-1 peptide adsorbed to HA [36]. Such conformations argue strongly against there being any stereochemical relationship between a folded structure of the peptide and an array of Ca^2+^ ions in the crystal lattice.

A synthetic peptide corresponding to the pOPAR sequence was generated, as was a nonphosphorylated version of the same sequence (OPAR). In our previous studies on the OPN-COM interaction, we validated our molecular-dynamics analysis by examining the ability of fluorescently labelled OPN peptide 220–235 (P3) to adsorb to COM crystals by confocal microscopy, and its effect on crystal growth habit by growing COM in the presence of peptide and determining crystal size by scanning electron microscopy [9]. Neither technique is feasible for HA, which typically forms much smaller crystals. Therefore, we studied the effects of OPAR and pOPAR on HA formation using a constant-composition/seeded-growth method.

Constant-composition growth of HA seed crystals, originally developed by Tomson and Nancollas [58], is the most rigorous quantitative method for studying HA formation. Because it involves growth of seed crystals, a much lower supersaturation can be used than is required for spontaneous nucleation of HA. Because a constant supersaturation is maintained by addition of Ca^2+^, PO_4_^3-^ and OH^-^ to replace those incorporated into the seed crystals, linear growth occurs. The constant-composition/seeded growth method has been widely used to study inhibitors of HA and other crystal phases [26], [74], [75], [76].

As previously noted by others, linear growth of HA seed crystals under constant-composition conditions only occurs after an initial period of non-linear growth [77], [78]. During the non-linear growth period, the seed crystals are growing at edges, kinks and screw dislocations. Once the edges and kinks are filled in, growth occurs only at screw dislocations [79].

In plots of linear growth rate against peptide concentration, the data fall on simple exponential decay curves, allowing us to calculate IC_50_ values for OPAR and pOPAR of 2.97 and 1.93 µg/ml, respectively. The small magnitude of this difference may seem surprising, in view of the abundant literature showing that phosphorylation of OPN is critical for its crystal-inhibiting activities (see Introduction). Even without a phosphate group, however, OPAR has a pI of 3.60, which our molecular-dynamics analysis predicts will result in strong interaction with the {100} face of HA (see Figure 2).

For purposes of comparison, we also performed constant-composition/seeded-growth analysis on peptides P3 and P0. The IC_50_ for P3, 1.48 µg/ml, is lower than those of OPAR and pOPAR, whereas the weak inhibitory activity of P0 meant that no IC_50_ value could be determined. The isoelectric point of P3 is 2.92. According to the relationship we have derived between pI and predicted {100}-face binding, P3 would be expected to be a strong inhibitor of HA growth. The isoelectric point of P0, 4.17, corresponds to a predicted peptide-crystal distance of approximately 1.4 nm, intermediate between the strongest (∼0.8 nm) and weakest (∼3 nm) OPN peptides (Figure 2). One might therefore expect that P0 would have stronger inhibitory activity than that measured by our constant-composition analysis. However, we have previously shown that differently phosphorylated proteins and peptides can adsorb equally well to COM crystals but vary widely in inhibitory activity [9], [22]. It may well be that fairly small differences in occupancy time on the crystal surface are crucial in determining whether or not step-pinning occurs. Also, the uptake of lattice ions measured in our constant-composition assay represents the growth of all faces present. A peptide of intermediate pI such as P0 may adsorb poorly to faces less basic than {100}.

We previously used a constant-composition method to study the inhibition of spontaneous (non-seeded) formation of HA by OPN phosphopeptides. This showed that peptides corresponding to sequences 41–52 (PQNSVpSpSEETDD) and 290–301 (SHELEpSpSpSSEVN) of rat bone OPN are more potent inhibitors of HA nucleation than peptide 7–17 (EFGpSpSEEKAHY) or 248–264 (IDpSQASSKApSLEHQpSHE) [29]. These peptides have isoelectric points of 2.09, 2.36, 3.70 and 3.93, respectively (calculated as described in Experimental Procedures). In agreement with the findings of the present study, therefore, the more-inhibitory peptides have lower isoelectric points than the less-inhibitory ones.

It is important to bear in mind that the IC_50_ values reported here reflect only one aspect of a peptide's HA-inhibiting activity. For OPAR and pOPAR, the inhibition curves reach an asymptote at a growth rate of zero. For P3, the asymptote is reached at approximately 20% of the control rate. Therefore, based on IC_50_ values, P3 is a stronger inhibitor than OPAR; based on maximal inhibitory effect, OPAR is the stronger inhibitor. It is not clear why some peptides can completely inhibit HA growth while others apparently cannot.

In conclusion, we have demonstrated the feasibility of using molecular dynamics as a screening technique to identify crystal-binding sequences in proteins. The sequences in rat bone OPN predicted to interact most strongly with the basic {100} face of HA have low isoelectric points due to the presence of aspartic acid, glutamic acid, phosphoserine and/or phosphothreonine residues. These sequences are also highly unordered, which may facilitate their interactions with Ca^2+^ ions of the crystal surface. In agreement with this model of protein-crystal interactions, we have synthesized highly anionic peptides based on sequences in OPN and shown that these are both unordered and potent inhibitors of HA growth.

## Supporting Information

Figure S1 Cicular dichroism spectropolarimetry of synthetic osteopontin peptides. Panels A and C: samples dissolved at 0.4 mM 100 mM NaCl, 10 mM KCl, 10 mM HEPES, pH 7.4. Panels B and D: samples dissolved at 0.4 mM in 150 mM NaCl, 500 µM Ca(NO_3_)_2_, 300 µM Na_2_HPO_4_, pH 7.4. Spectra were collected on a Jasco J-810 spectropolarimeter at 37°C with a step size of 0.5 nm and a scan speed of 100 nm/min, using a 0.1-mm path-length cell.
(14.63 MB TIF)

Movie S1 Video of pOPAR peptide adsorption to the {100} face of hydroxyapatite. Colour scheme as described in Figure 5.
(1.62 MB MPG)
